# Does Unilateral Hearing Loss Impair Working Memory? An Italian Clinical Study Comparing Patients With and Without Hearing Aids

**DOI:** 10.3389/fnins.2020.00905

**Published:** 2020-09-08

**Authors:** Antonio della Volpe, Valentina Ippolito, Dalila Roccamatisi, Sabina Garofalo, Antonietta De Lucia, Valeria Gambacorta, Fabrizio Longari, Giampietro Ricci, Arianna Di Stadio

**Affiliations:** ^1^Otology and Cochlear Implant Unit, Department of Otolaryngology, Santobono-Pausilipon Children’s Hospital, Naples, Italy; ^2^Psychology Faculty, Università Telematica Internazionale Uninettuno, Rome, Italy; ^3^Department of Otolaryngology, University of Perugia, Perugia, Italy; ^4^Neuroinflammation Laboratory, Queen Square Neurology, University College London, London, United Kingdom

**Keywords:** unilateral hearing loss, hearing aids, speech rehabilitation, working memory, hearing loss, bone anchored hearing device

## Abstract

Working memory (WM) function can be reduced in patients suffering from unilateral hearing loss (UHL) and can affect their academic performance. We aimed to compare the WM abilities of three categories of children with UHL: patients implanted with hearing aids (HAs), patients receiving a bone-anchored hearing implant (BAHI), and subjects who did not receive hearing devices. A randomized clinical study, in which 45 children (mean age: 9.5 years) were evaluated by pure tone audiometry (to identify the side and the severity of the UHL), was conducted in a tertiary referral center. Patients were simply randomized into three groups: (1) children without HAs (No-HA group), (2) patients with a (digital) HA (HA group), and (3) children with a BAHI (BAHI group). Their working and short-term memories were studied in both noisy and silent conditions at the recruiting time (T0, baseline) and 6 months after (T1) the treatment. Statistical analyses were performed to analyze the variances between T0 and T1 within each group and between the three groups. The No-HA group improved its T1 WM scores in silence (*p* < 0.01), but not in noise. The HA and BAHI groups showed statistically significant variances of T1 WM in noise (*p* < 0.01 and *p* < 0.01, respectively). The HA and BAHI groups did not show statistically significant variances compared to T1. Our results suggest that hearing devices (HA and BAHI) in children with sensorineural UHL (SUHL) can improve WM capacity in noise. We speculate that bilateral hearing capacity might improve the quality of life of this population, especially during everyday activities where noise is present.

## Introduction

The central auditory pathway is bilaterally stimulated by the ears due to crossing fibers, so a unilateral hearing function could modify sound perception (Peterson and Hamel, 2019). When both ears (afferent pathways) work normally (healthy subject), sounds reach the central auditory area reinforced (Popper and Fay, 2019) due to a summation of the auditory stimulus. The reinforcement of the stimulus positively affects the left brain—responsible for speech understanding and production (Knösche et al., 2002)—and it allows correct understanding of the meaning of speech and development of normal language skills, especially in children (Di Stadio et al., 2018).

The literature has shown that restoring bilateral hearing functions in children can improve speech perception (Glasberg and Moore, 1989; Mok et al., 2010; Di Stadio and Lazzaro, 2015; Di Stadio et al., 2018), language skills (Lieu et al., 2010; Di Stadio and Lazzaro, 2015; Fitzpatrick et al., 2019), and memory functions (Lyxell et al., 2003; Ning et al., 2013; Di Stadio et al., 2018). The improvement of memory function is related to the increase in attention, and the latter is better when the subject can hear correctly with both ears (Corballis, 2014; Bartolomeo and Seidel Malkinson, 2019). Other studies showed that children with unilateral hearing loss (UHL) can benefit from bone-anchored hearing implant (BAHI) rehabilitation especially at school, as shown by the improvement of their dictation skills (Di Stadio et al., 2018). Dictation skills need to be supported by memory [short-term memory (SM)] functions, so their improvement is directly correlated with the increase of memory performance; the hearing device allowed UHL children to improve their working memory (WM) scores up to the same results as healthy children, and this could explain the observation of Di Stadio et al. (2018).

There is only one study that analyzed the impact of bilateral hearing restoration on the memory performance of UHL children, only investigating patients exclusively treated by BAHI. Even today, there is very little information on the effect of different hearing aids on memory abilities. We therefore aimed to compare the effects of non-use vs. use of hearing aids (HA or BAHI) on memory performance in a sample of children suffering from UHL of varying severity.

## Materials and Methods

The study was conducted at the Cochlear Implantation Centre of the Santobono-Pausilipon Children’s Hospital in Naples (Italy) from January to September 2019, and it was performed according to Helsinki rules for human study. The protocol was approved by the internal review board (IRB) of the hospital, without issuing an authorization number in respect to national regulations for these types of studies. Before including the children in the study, all parents needed to sign a written consent, in which strengths and weaknesses of each treatment were discussed.

Forty-five children [24 males and 21 females, average age 9.5 years (*SD*: 2.8)] affected by congenital [70.8% of children (32 subjects) suffered from HL connexin 26-related, 19 heterozygous (60%) and 13 homozygous mutation, 25% from cytomegalovirus (CMV)-related HL, and 4.2% of mitochondrial disease-related HL] sensorineural UHL (SUHL) that ranged from mild (26–40 dB) to severe (90 dB) and who presented a normal hearing threshold on the contralateral side were enrolled in the study (Table 1).

**TABLE 1:** The table summarizes demographic details of the patients included in the study.

	No-HA	HA	BAHI
Age	10 ± 3.43 (SD)	10 ± 2.41 (SD)	9 ± 2.17 (SD)
Gender	13 patients:	16 patients:	16 patients:
	7 male- 6 female	7 male- 9 female	10 male- 6 female
Hearing loss PTAv T0*	63.5 ± 8.18 (SD)	63.5 ± 12.6 (SD)	63.7 ± 10 (SD)

*Please see additional details of patients’ auditory thresholds Table 4. No-HA, no hearing aids; HA, hearing aids; BAHI, bone anchored hearing implants; SD, standard deviation; PTAv, pure tone average.

All children performed CT scan to evaluate the presence of temporal bone abnormalities; if some abnormalities were identified, the patients were excluded from the study.

The inclusion criteria were first diagnosis of SUHL, patient never rehabilitated by speech therapy and/or hearing device, Italian mother-tongue, and without known/evident intellective deficit.

All patients were tested twice, at baseline (T0) and 6 months after their first control (T1). They underwent a bilateral auditory pure tone test (PTA) at T0 in order to evaluate their hearing capacity and to confirm the presence and the side of the SUHL (details below) and then at T1. A Speech Perception Test (SPT) was also performed at T0 and T1 in all children.

Three groups were defined: group 1 (control group) in which patients with SUHL were not treated with hearing aid (No-HA group), group 2 that included patients who were treated with hearing aid (HA), and group 3 in which patients’ SUHL was treated by a bone conduction system (BAHI group).

The children were randomized by computer software to be assigned to one of the three groups and then to be treated as indicated by the relative group. The computer generated the random numbers that were used for the simple randomization. For a correct distribution in each group, the patients were separately randomized according to their HL severity; first, patients with mild SUHL were randomized, then the ones with more severe forms up to the ones with profound SUHL. Despite this initial randomization, we then revised the assignation of the children to one or another group to avoid undertreatment or overtreatment of SUHL, so we cannot define this study as a real randomized study.

The randomization by different severities of SUHL was possible because the children had one ear with normal hearing threshold. Children with mild SUHL had an HL in the range in which BAHI could be an effective treatment.

Patients in group 1 were untreated for speech rehab, while patients in groups 2 and 3 were trained by a speech therapist to optimize the use of their hearing aid. Patients with BAHI performed speech rehab once their prosthesis was osteointegrated. However, regardless of which group they were assigned to, all children underwent cognitive and learning rehabilitation.

All patients in group 2 used Xceed Play 1 BTE SP^1^ as hearing device. This digital device, specifically intended for children, is designed for treatment of sensorineural hearing loss (SNHL) in the severity range between 30 and 110 dB. The device covers a frequency range between 100 and 6,500 HZ, has an OSPL90 with maximum peak at 143 dB SPL and 135 dB SPL at 1,600 Hz, and allows a maximum of 83 dB at gain peak and 75 dB at 1,600 Hz. Its harmonic distortion at 70 dB SPL is 4% at 500 Hz, < 2% at 800 Hz, and < 2% at 1,600 Hz. The device entry noise level was 18 dB SPL omni and 32 dB SPL directional. Finally, it has an integrated receiver with a power of 2.4 GHz and is FM compatible. The hearing aids were used to compensate the hearing deficit. Hearing aids had to be worn all day from wake-up to bed time.

Patients in group 3 were implanted with BAHA^®2^. The BAHA^®^ was implanted percutaneously as previously described by Ricci et al. (2019) by the same surgeon with decades of experience in hearing devices. In this group, the BAHI was used as a CROS device.

The memory abilities were evaluated in all children at T0 before randomization both in silent and in noisy conditions (T0) and at T1–6 months after the hearing rehabilitation or the application of a hearing prosthesis. This 6-month follow-up was necessary to allow a correct osteointegration of the BAHI (3 months in all our patients) and adequate speech rehab once the prosthesis was implanted. A memory evaluation included testing both the WM and the SM, as detailed below. All tests were conducted by a speech therapist and a psychologist together; both professionals had more than 10 years of experience in their respective field.

### Details on Clinical Investigation

#### Pure Tone Audiometry

The auditory tests were performed differently at T0 and T1. At T0, the patient was tested by earphones in a silent cabin. At T1, children were analyzed in free-field using speakers because they were wearing a hearing prosthesis. The T1 auditory test was performed with the patient seated in the center of the cabin where two speakers at 45° were placed one on each side of the patient. A pulse-tone was emitted by the speaker on the side of the ear that had to be tested while a white-noise sound (masquerading sound) was sent by an insert located on the opposite side. This procedure was performed bilaterally. The sound stimulation started from 10 dB, with increases of 10 dB and decreases of 5 dB, to confirm the sound perception. The impulse for each frequency tested (250, 500, 1,000, 2,000, 4,000, 6,000, and 8,000 Hz) was sent to the studied ear three times, following the method described above. The masking signal (white noise range frequency 2–16 kHz) was sent at 50 dB and the testing signal at 20–30 dB on the ear in which we were evaluating the hearing threshold. Pure tone average (PTAv) was calculated by summing the results of pure tone audiometry (PTA) thresholds at 500, 1,000, and 2,000 Hz and dividing the total by 3.

#### Speech Perception Test

The test was performed in the same conditions as PTA. A list of simple words (Table 2) was used in place of the pulse tone and the cocktail party sound (several adult talkers) replaced the white noise sound (masquerading sound). The masking signal was sent at 50 dB, and the testing signal at 20–30 dB on the ear in which we were evaluating the hearing threshold. The test was performed with the same procedure described for PTA. SPT was measured with an average percentage, with values from 0% total absence of perception up to > 95% indicating perception of all proposed words.

**TABLE 2:** The table shows the list of Italian words used to perform speech perception test in Italian.

List of words used for speech perception test in children			
mamma	camion	lupo	albero
scuola	letto	spada	quadro
nonno	frigorifero	occhio	chiodo
orologio	fiori	cesto	stelle
uno	giornale	televisione	regina
macchina	sedia	occhiali	treno
pipa	famiglia	farfalla	due
forchetta	limoni	cane	acqua
casa	gatto	bagno	bicicletta
capelli	sci	barca	pantaloni

*This words’ list is specifically designed for children and it was the only used for testing all patients.*

#### Working Memory Testing

The WM actively manages received information. It allows us to understand the meaning of a sentence even if the meaning of each individual word is not known (Baddeley, 1981). Evaluation of the WM was performed with PROMEA battery tests. Subjects were asked to repeat one meaningless word at a time; the word (bisyllabic to multisyllabic) consisted of an alternation of vowels and consonants with sounds very phonologically similar to known (existing) Italian words (Vicari, 2007). A “non-word” sentence is a sentence composed of pseudo-words, which are words that have a sound similar to words in the (Italian) language but which, in fact, have no meaning [e.g., “sasta” sounds similar to “pasta” (Vicari, 2007) but does not mean anything]. A subject with a properly functioning WM will correct the non-word “sasta” to “pasta,” indicating an understanding of the meaning of the sentence as a whole. All tests were performed in both silent and noisy (cocktail party noise) conditions (Hutcherson et al., 1979). Test scores were calculated by dividing the number of correct answers by the total number of questions, which was 39, as suggested by Vicari in the book *Tests for Memory and Learning in Childhood*. The memory test was standardized for children aged 5–11 years (Bisiacchi et al., 2005; Vicari, 2007). The final score was calculated in percentages as follows: 0–25% severe WM deficit; 26–50% moderate WM deficit; 51–75% adequate WM; and 76–100% excellent WM.

#### Short-Term Memory Testing

The SM stores information for a brief period of time, typically 10−15 s (Baddeley and Patterson, 1971). Evaluation of the SM was performed by asking the subjects to repeat the words exactly as they were heard. This test was performed under silent conditions only, as noise may affect SM function and impair the subject’s ability to hear a non-word. We chose repetition of phonologically similar words (similarity effect) to evaluate and stimulate the phonological loop, the efficiency of which is strongly related to the auditory function. The test was performed by presenting six blocks (Glasberg and Moore, 1989; Knösche et al., 2002; Di Stadio and Lazzaro, 2015; Di Stadio et al., 2018; Peterson and Hamel, 2019; Popper and Fay, 2019) of words not commonly used (low-use frequency) in sequence. Each block of words contained a sequence of bisyllabic words, i.e., “luna-topo,” which were presented to the patients five times consecutively at intervals of 2–3 s. The test started by proposing a list of two words, then three, four, etc. If the patient correctly repeated 3/5 sequences of heard words, the examiner went ahead to the next span level. The answers to each block identify a different level of span, from 1 to 6. Final scores of span level could range from 1 to 6, where scores between 1 and 3 indicated an SM deficit, and scores ≥ 4 indicated normal SM function. This SM test was standardized for children aged between 5 and 11 years (Bisiacchi et al., 2005).

### Data Analysis

Because all patients were analyzed after 6 months from their first control (T0), the three groups did not present statistically significant differences in observation time; the variable “time” was not considered due to this reason. All WM scores (groups 1, 2, and 3) collected at T0 in noisy and in silent conditions and at T1 in the same conditions were compared using a one-way ANOVA test and by Holm−Bonferroni method. The same analysis was performed to compare SM scores at T0 and T1 in the three groups (groups 1, 2, and 3). We also analyzed the difference between T0 and T1 considering T1 as dependent variable by using a two-tailed *t-*test. Our sample included patients affected by SUHL on different sides, so we also evaluated the differences between the right and left side. For all tests, the level of significance was set to 0.05. The statistical analysis was performed with Stata^®^ and was supervised by an expert in biostatistics and epidemiology.

## Results

### General

The time elapsed (days) between the first (T0) and the second (T1) follow-up in the three groups was: No-HA 180 (*SD*: 3.8), HA 180.3 (*SD*: 3.4), and BAHI 180.6 (*SD*: 3.7).

Group 1 (No-HA) included four patients with mild HL, five with moderate HL, three suffering from moderate to severe HL, and one with severe SUHL. In group 2 (HA), eight patients suffered from mild SUHL, five with a moderate form, two affected by moderate–severe SUHL and one with severe SUHL. In group 3 (BAHI), we observed three patients affected by mild SUHL, two with a moderate form, two with moderate–severe SUHL, and nine with severe SUHL.

Table 3 shows the results of PTA and SPT at T0 and T1 in the three groups.

**TABLE 3:** The table shows the results of PTA and SPT in the three groups at T0 and T1.

	No-HA	HA	BAHI
PTAv T0	63.5 ±8.18 (CI 95%: 55–80)	63.5 ±12.6 (CI 95%: 45–90)	63.7 ±10 (CI 95%: 55–90)
PTAv T1	57 ±8.2 (CI 95%: 45–70)	23.2 ±6.5 (CI 95%: 15–35)	22 ±3.3 (CI 95%: 20–30)
SPT T0	45% ±9 (CI 95%: 30–65)	45.7% ±12 (CI 95%: 35–70)	42.7% ±3 (CI 95%: 40–45)
SPT T1	52% ±8 (CI 95%: 40–65)	91.5% ±8 (CI 95%: 75–100)	93% ±6 (CI 95%: 80–100)

*No-HA, no hearing aids; HA, hearing aids; BAHI, bone anchored hearing implant; PTAv, pure tone average; SPT, speech perception test.*

**FIGURE 1:**
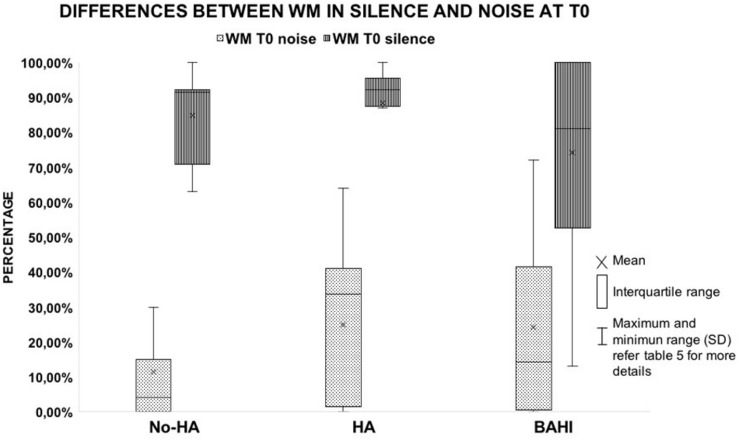
The image shows the differences observed in the working memory score between the test performed in silent and noisy conditions at T0 in the three groups. All the horizontal lines in the box divide II and III quartiles.

Twenty-five subjects [15 males and 10 females, mean age: 9.3 years (*SD*: 2.8)] suffered from right-sided SUHL, while 20 subjects were affected by left-sided SUHL [five males and seven females, mean age: 10 years (*SD*: 2.8)]. A statistically significant variance was identified between the WM scores in noise and silence at T0 in all three groups (*p* < 0.00001; BH subgroup 1: *p* < 0.01; subgroup 2: *p* < 0.01; subgroup 3: *p* < 0.01); no statistically significant differences were identified between the groups at T0 (Table 4 and Figure 1).

**TABLE 4:** Summary of PTAv at T0, WM at T0 (silence and noise), SM at T0, WM at T1 (silence and noise), and SM a T1.

Group	Side of UHL	Hearing thresholds PTAv T0 (dB)	WM T0 noise	WM T1 noise	WM T0 silence	WM T1 silence	SM T0	SM T1
*No-HA*	Right	55	0%	0%	91.6%	92%%	4	4
*No-HA*	Right	80	0%	0%	100%	100%	2	2
*No-HA*	Right	35	66%	66%	87%	90%	4	4
*No-HA*	Right	40	29.1%	30%	100%	100%	5	5
*No-HA*	Right	60	15%	15%	92.3%	92.5%	4	4
*No-HA*	Right	55	8%	10%	92%	92%	4	4
*No-HA*	Right	70	8%	9%	80%	80%	2	2
*No-HA*	Right	55	4%	8%	92%	92%	4	4
*No-HA*	Right	40	30%	30%	100%	100%	4	4
*HA*	Right	55	41.6%	80%	100%	100%	3	3
*HA*	Right	90	0%	46%	92%	92%	4	4
*HA*	Right	35	45%	83%	87%	87%	4	4
*HA*	Right	40	37.5%	70%	100%	100%	4	4
*HA*	Right	70	12%	88%	90%	90%	3	3
*HA*	Right	40	30%	84.6%	92.3%	92.3%	4	4
*HA*	Right	35	41%	100%	91.6%	91.6%	4	4
*BAHI*	Right	55	0%	80%	80%	85%	2	3
*BAHI*	Right	40	41.6%	95%	41.6%	41.6%	3	3
*BAHI*	Right	60	12.5%	33%	100%	100%	4	4
*BAHI*	Right	85	37.5%	79.4%	50%	60%	4	4
*BAHI*	Right	90	6%	92.3%	92.3%	92.3%	2	4
*BAHI*	Right	75	41%	89.4%	76.9%	78%	3	3
*BAHI*	Right	90	0%	60%	13%	15%	3	3
*BAHI*	Right	90	46%	79.4%	82%	83%	3	3
*BAHI*	Right	90	0%	33%	61%	62%	4	4
*No-HA*	Left	35	0%	0%	63%	64%	3	3
*No-HA*	Left	55	0%	0%	70.8%	71%	4	4
*No-HA*	Left	65	8%	10%	80%	82%	2	2
*No-HA*	Left	35	0%	0%	63%	64%	3	3
*No-HA*	Left	50	4%	5%	92%	92%	4	4
*No-HA*	Left	55	0%	4%	70.8%	72%	4	4
*HA*	Left	35	64%	100%	94.8%	95%	4	4
*HA*	Left	55	2%	61%	92%	92%	4	4
*HA*	Left	40	2%	66%	87.50%	89%	4	4
*HA*	Left	60	0%	30%	48%	50%	3	3
*HA*	Left	55	0%	95%	97.4%	98%	4	4
*HA*	Left	45	37.5%	79%	70%	70%	4	4
*HA*	Left	40	38%	58%	95%	95%	5	5
*BAHI*	Left	35	72%	100%	100%	100%	3	3
*BAHI*	Left	45	16%	62.5%	100%	100%	4	4
*BAHI*	Left	90	0%	60%	13%	15%	3	3
*BAHI*	Left	65	41%	89.4%	76.9%	76.9%	3	3
*BAHI*	Left	75	2%	70%	100%	100%	4	4
*BAHI*	Left	35	72%	100%	100%	100%	3	3
*BAHI*	Left	80	2%	70%	100%	100%	4	4

*No-HA, no hearing aids; HA, digital hearing aids; BAHI, bone anchored hearing implant; WM, working memory; SM, short-memory; PTAv, pure tone average.*

**FIGURE 2:**
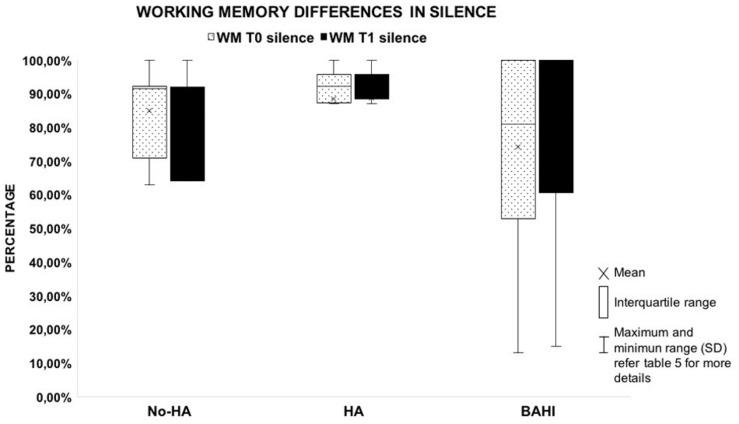
The plot shows the absence of variances between the working memory score collected in silent condition by comparing T0 and T1. The graph compares the three groups. All the horizontal lines in box divides II and III quartiles.

**FIGURE 3:**
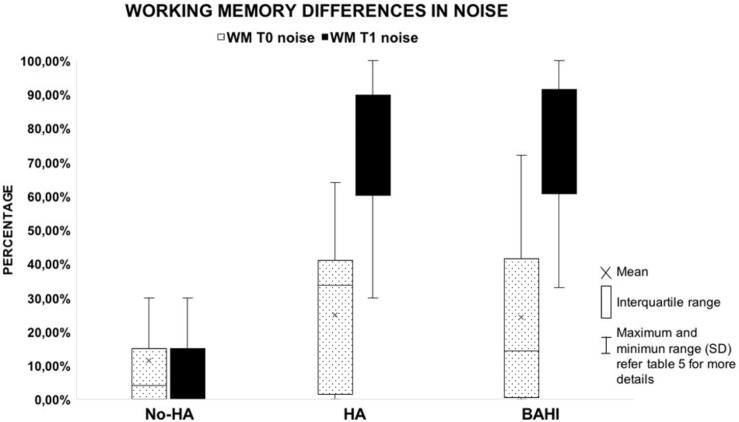
The graph illustrates the recovery of working memory in the noisy condition. Despite the fact that patients with no hearing aids (HA) improved their scores, the results failed to reach statistical significance. All the horizontal lines in the box divides II and III quartiles.

**FIGURE 4:**
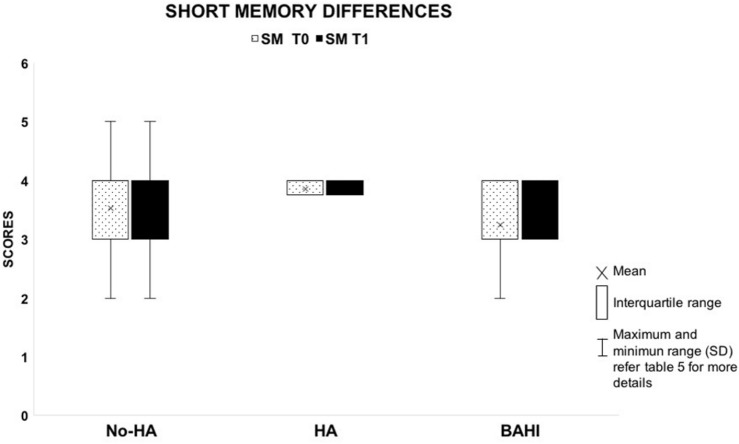
The image shows the absence of variances between T0 and T1 in the short-term memory scores between the three groups.

No statistically significant variance was observed in silence between the WM scores at T0 and T1 in group 1 (No-HA; BH: *p* = 0.3) or in noise (Figures 2, 3). Group 2 (HA) presented statistically significant variances in noise comparing T0 and T1 (HB: *p* < 0.01), but no statistically significant differences were observed in silence. A similar finding was observed in group 3 (BAHI; HB: *p* < 0.01; Figures 2, 3). Statistically significant differences in the WM scores were observed at T1 (silence and noise) between patients in group 1 (No-HA) and subjects with hearing devices (HA and BAHI; HB: *p* < 0.01 and *p* < 0.01, respectively; Figures 2, 3). No statistically significant differences were observed in groups 2 and 3 between the WM scores at T1. The side of SUHL (right vs. left) did not affect the WM scores at T0 and T1 (no statistically significant result). No statistically significant variances were observed by comparing the SM scores at T0 and T1 between groups and within each group (Figure 4).

The analysis of data, considering T1 as a dependent variable of T0, showed in the No-HA group a statistically significant difference in the WM scores both in noise (*t*-test: *p* = 0.01) and silence (*t*-test: *p* = 0.01), but not statistically significant values in SM (*t*-test: *p* = 1). HA and BAHI groups presented statistically significant differences in the WM scores in noise, respectively *p* < 0.00001 and *p* < 0.00001. No statistically significant differences were observed in silence in HA (*t*-test: *p* = 0.09) and BAHI (*t*-test: *p* = 0.05) patients. SM values were not statistically significantly different in both groups (HA: *t*-test: *p* = 1 and BAHI: *p* = 1) (Table 5).

**TABLE 5:** The table summarizes SD and mean in each group of WM at T0 and T1 both in silence and noise, and SM at T0 and T1.

Group	WM T0 (%) noise	WM T1 (%) noise	WM T0 (%) silence	WM T1 (%) silence	SM T0	SM T1
*No-HA*	10.2 ±0.2	12.4 ±0.2	86.2 ±0.1	84.5 ±0.1	3.5 ±0.9	3.5 ±0.9
*HA*	21.3±0.2	74 ±0.2	86 ±0.1	88 ±0.1	3.8 ±0.5	3.8 ±0.5
*BAHI*	25 ±0.3	73 ±0.2	74.5 ±0.3	76 ±0.3	3.2 ±0.7	3.4 ±0.5

Finally, no statistically significant variances were observed in the SM scores (T0 and T1) between right- and left-sided SUHL.

## Discussion

Our study showed that the restoration of a bilateral hearing function in UHL subjects could improve WM functions, especially in noisy condition, and that this improvement was independent of the device used to rehab the auditory capacity, of the side affected by HL and of the severity of HL.

These current results reinforce other recent observations (Di Stadio et al., 2018), in which the authors showed that children with UHL rehabilitated by BAHI could improve their WM capacities up to the levels observed in healthy children. In addition, these positive results were more consistent when the memory functions were tested in noisy condition.

The analyses on T1 as dependent variable of T0 confirmed that HA and BAHI had improved the scores of WM in noise but not in silence and that the scores of SM remained substantially the same at the two follow-ups (T0-baseline and T1-6 months after baseline). Interesting data were that No-HA children statistically improved their WM scores in silence but not in noise; in the latter, we instead observed a statistically significant worsening of the WM capacities. We speculated that the difference observed in noise scores between the three groups could be related to three factors: (1) use of hearing device, (2) speech rehabilitation, and (3) cognitive and learning rehabilitation. In fact, in the No-HA group, the cognitive and learning rehab alone negatively impacted on the WM abilities in noise as shown by the statistically significant worsening of the scores that we observed.

We also noted that, regardless of the severity of UHL, patients with hearing devices improved their PTA and SPT on comparing T0 and T1 (Table 3); this positive effect of hearing rehabilitation on speech perception in children with UHL—regardless of HL severity—was already shown by Purcell et al. (2016). In addition to the hearing device, also the speech rehabilitation performed by the children in groups 2 and 3 could have been helpful in improving PTA and SPT capacities.

Bishop et al. (2017) showed that the increase in speech perception could improve WM performance; the observed memory recovery could be related not only to the improved speech perception but also to an increased perception of external stimuli (Arndt et al., 2017) since both conditions stimulate memory functions (Cardon and Sharma, 2013). The mechanism which drives the improvement of WM is based on the amelioration of attention (Cherubini, 2012); the restoration of bilateral hearing allowed the patient to capture information more correctly and to better select which of these could be relevant or not (Cherubini, 2012). Thanks to the better hearing capacity, patients were able to strategically shift the focus of attention between maintenance and processing over time and in the most appropriate ways (Cherubini, 2012). However, we cannot exclude that speech rehabilitation also had positively impacted on WM function by partially influencing the results observed. In fact, both hearing restoration and rehabilitation performed by the children could have stimulated their brain plasticity as previously shown by Kolb et al. (2017). These authors focused their analyses on brain plasticity, and they found that children’s brains were extremely sensitive to changes in external stimuli (Kolb et al., 2017), so the restoration of bilateral hearing function had positively impacted on their brain maturation (Cardon and Sharma, 2013; Kolb et al., 2017). Furthermore, as previously shown by Pisoni (1973), the correct identification of words positively impacts on memory functions.

Although both are very efficient in rehabilitating auditory functions, the hearing devices that we used in our study worked in different ways. The digital device improved the sound perception by increasing the volume of the incoming sound (Levitt, 2007); the BAHI, on the other hand, stimulated the cochlea by a vibratory stimulation activating cochlea hair cells and by exciting the basilar membrane through the inertia of the inner ear fluid and the compression and expansion of the inner ear space (Stenfelt, 2015). In addition, the BAHI could retrogradely stimulate the perilymph through the vibration of cerebrospinal fluid (CSF) (Sohmer et al., 2000). Nevertheless, our results showed that, regardless of the hearing device, bilateral hearing function improved the memory performances. However, considering that children with BAHI underwent delayed speech rehab compared to patients with a hearing device, we could affirm that this type of hearing rehabilitation allowed better auditory performance; in fact, children achieved an improvement of WM performance as good as the one observed in patients with hearing aids but with fewer speech rehabilitation training sessions.

The benefits achievable by bilateral hearing functions have already been described (Krishnan and Van Hyfte, 2016); this restoration impacts positively on memory functions (McCoy et al., 2005; Härkönen et al., 2015; Di Stadio et al., 2018) and improves word recollection performances (Skinner, 1957) by helping school-age children in the execution of certain tasks [e.g., dictation (Di Stadio et al., 2018)]. On the contrary, patients affected by untreated UHL can suffer from behavioral consequences (Brookhouser et al., 1991) and present unsatisfactory academic performances (Dancer et al., 1995). A correct hearing function, especially in noise (silence is rarely present in children’s classrooms), is fundamental to avoid developmental delays (Brookhouser et al., 1991; Härkönen et al., 2015).

Although there is still some controversy on the use of hearing devices to support children with UHL (McKay et al., 2008; Bagatto, 2020)—some authors have indicated that a reduction of hearing performance in noise may be related to distraction on the side in which UHL was treated by hearing device (Lewis et al., 2016)—our results suggest that bilateral hearing functions could be necessary to improve the memory abilities of UHL children, particularly in everyday life rich in surrounding noise (Di Stadio et al., 2020). In fact, regardless of the severity of their HL, all our children presented a worsening of WM scores comparing their answers in silence and noise at T0, meaning that noise negatively impacts on memory performances. At the end of our study, we noted the improvement of the WM scores in all children (regardless of the severity of their HL), although the highest gains were identified in the most severe cases of UHL (T0 vs. T1). This result is in contrast with the idea that a hearing device in UHL can negatively impact on a child’s performance (Bagatto, 2020).

Based on our results, we suggest that a hearing device should always be used to avoid impairment of scholastic performance, regardless of the severity and the side of the HL.

An important aspect to emphasize was the strong impact that speech rehabilitation had on maximizing the benefit of the hearing device. The speech therapist helped patients with hearing devices to focus their attention on what could be better perceived by wearing a prosthesis. This aspect was very helpful in better managing the distress related to wearing the device and improving patient compliance. The speech rehabilitation helped subjects to correctly focus their attention on the new external stimuli. The speech therapist not only increased the compliance of children in using the prosthesis, but she also supported parents in the management of this new situation, i.e., answering the questions related to the use of the device. The speech training was useful for children and parents/guardians. The latter, by understanding the efficacy of the prosthesis, strongly supported their children’s hearing aid compliance.

The study presents some limitations. Firstly, the right-sided UHL group was slightly larger than the left-sided UHL group, and this may induce bias due to the unbalanced distribution of subjects. Secondly, the tests used to evaluate speech perception and memory functions were designed exclusively for Italian speakers, and other authors could obtain different results using other tests. In addition, we were focused on memory only and the attention of the patients could only be indirectly evaluated by looking at the results of the memory tests. Furthermore, we did not analyze if sound localization could impact on memory function, although we did not find differences comparing patients with hearing device on the right side with the ones in the left one. Additional studies investigating the effect of sound localization on memory function would be necessary.

Our patients’ selection, in which we excluded patients affected by temporal bone abnormalities, might have determined a bias; in addition, the study has been performed on a small sample and other researchers might obtain different results studying a larger sample. In fact, our population of children could not reflect the broader universe of children with UHL.

Another limitation, related to an ethical reason, was the slight unbalance between untreated patients (No-HA: 13 subjects) and patients treated with hearing aids (HA: 16 people and BAHI: 16 patients). Moreover, because of ethical and clinical reasons, group 3 (BAHI) included more patients with severe SUHL than the other two groups; in fact, restoration of an efficient hearing threshold was the main goal of this treatment. Finally, because of the use of a hearing device, auditory tests were differently performed at T0 (no hearing device) and T1 (wear hearing device), and this difference in tests might have an impact on the results of the study. However, this limitation is present in all studies of this type.

## Conclusion

Hearing restoration *via* hearing aids/BAHI or hearing therapy should be suggested as the treatment of patients suffering from UHL, given that bilateral hearing stimulation may positively impact on the WM processes, especially in noisy conditions (Di Stadio et al., 2020). In fact, non-rehabilitated UHL could lead to a reduction in memory performance during school activities (rich in noise) which could negatively impact on academic performance. We suggest rehabilitation by hearing device for all children affected by UHL, regardless of the severity of their HL, to avoid possible deficiencies in school performances.

## Data Availability Statement

The raw data supporting the conclusions of this article will be made available by the authors, without undue reservation.

## Ethics Statement

The studies involving human participants were reviewed and approved by Santobono-Posilipon IRB. Written informed consent to participate in this study was provided by the participants’ legal guardian/next of kin.

## Author Contributions

ADS contributed to the study design, analysis of data, interpretation of data, state of conclusion, supervision of the study, and article writing. AV contributed to the analysis of data, state of conclusion, and data collection and provided substantial contribution in article writing. VI contributed to the data collection and interpretation of data and provided supporting contribution in article writing. SG and ADL contributed to the data collection and data interpretation. VG and FL contributed to supporting data collection. GR contributed to criticism and provided supporting contribution in writing. All authors contributed to the article and approved the submitted version.

## Conflict of Interest

The authors declare that the research was conducted in the absence of any commercial or financial relationships that could be construed as a potential conflict of interest.

## Footnotes

1 www.oticon.com

2 www.cochlear.com
